# Avoiding misdiagnosis of duodenal papilla adenocarcinoma in a situs inversus totalis patient *via* laparoscopic pancreaticoduodenectomy: A rare case report

**DOI:** 10.3389/fsurg.2022.1058580

**Published:** 2023-01-06

**Authors:** Hao Liang, Ya-kun Wu, Wu Pang, Ming-liang Chen, Yu Zhu

**Affiliations:** Department of Hepatobiliary Surgery, Suining Central Hospital, Suining, China

**Keywords:** situs inversus totalis, laparoscopic pancreaticoduodenectomy, misdiagnosis, duodenal papilla adenocarcinoma, case report

## Abstract

Situs inversus totalis is a rare congenital anatomical anomaly that causes some difficult problems for surgeons when performing an operation. However, without histopathology specimens from surgery, a misdiagnosis of cancer may be unavoidable, in addition to affecting the improvement of prognosis. This study reports a rare patient with situs inversus totalis who presented with the main complaints of pruritus and vague abdominal pain. She was first misdiagnosed with cholangiocarcinoma and was finally diagnosed with duodenal papilla adenocarcinoma *via* laparoscopic pancreaticoduodenectomy. Situs inversus totalis was not a contraindication for surgery. Skilled surgeons and complete preparation during the perioperative period are two important keys to successful surgeries. Performing laparoscopic pancreaticoduodenectomy for patients with situs inversus totalis to avoid misdiagnosis of cancer and tailor appropriate therapy plans is cost-effective.

## Background

Situs inversus of viscera is a congenital anatomical anomaly, and patients with situs inversus are also called “mirror people”. The incidence of situs inversus is very low, from 1:10,000 to 1:20,000 (1). The reversal of thoracic and abdominal viscera or vessels in patients with situs inversus can be partial or complete (called situs inversus totalis, SIT). Simple SIT without multiple malformation syndrome does not shorten a person's life, and many patients with SIT are never even diagnosed throughout their lives. Those patients can live a normal life similar to people without SIT.

In patients with SIT, there are many challenges for surgeons in performing some complex operations, especially laparoscopic pancreaticoduodenectomy (LPD), when cancer is suspected. However, without histopathology specimens from surgeries, the risk of misdiagnosis of cancer increases, and the corresponding treatment may be inaccurate, eventually leading to a poor prognosis for SIT patients.

Some previous studies have reported attempted surgical treatments in patients with SIT, from simple laparoscopic cholecystectomy (2) to laparotomic resection of the pancreatic body and tail (3). However, the safety of performing LPD in the average patient is still questioned, et al. one in patients with SIT. Currently, few studies have reported on patients with SIT receiving LPD therapy and have noted the importance of surgery to avoid misdiagnosis of cancer in those patients (4).

This study reports a patient with SIT who was originally misdiagnosed with cholangiocarcinoma. After receiving LPD treatment, the patient was eventually diagnosed with adenocarcinoma of the duodenal papilla.

## Case report

A 50-year-old, right-handed woman was admitted to the Department of Hepatobiliary Surgery of the hospital because of main complaints of pruritus and vague abdominal pain.

Two years before this admission, the patient was seen by an ophthalmologist and was diagnosed with chronic dacryocystitis. After receiving lacrimal duct intubation therapy, she felt well and left the hospital. One year after this treatment, with a main complaint of vaginal bleeding, the patient was diagnosed with dysfunctional uterine bleeding. Interestingly, the results of computed tomography (CT) of the chest and abdomen revealed that this patient had SIT. Without contraindications, she underwent endometrial ablation therapy and left the hospital a few days later.

Approximately 1 month before admission, the patient visited a dermatologist with the main complaint of pruritus and received a local glucocorticoid treatment for the skin, but the symptoms remained. Approximately 10 days before admission, she complained of vague abdominal pain and terrible pruritus with jaundice.

There was no history of hepatitis, no known allergies to medications, and no family history of SIT or exposure to toxins. On examination, the patient was well groomed, pleasant and cooperative. There was jaundice of the sclera and tenderness in the upper abdomen. The heartbeats were heard best on the right side of the body.

**Figure 1:**
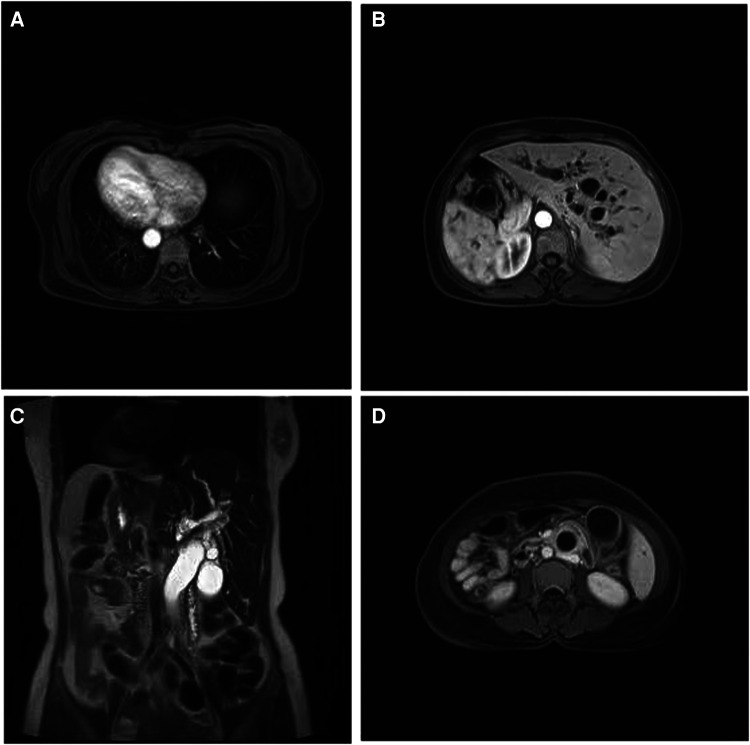
Abdomen MRI findings of the female with situs Inversus totalis. (**A**) Inversus of heart; (**B**) inversus of abdominal viscera (**C**) vine-tree-like dilatation of the bile ducts; (**D**) thickening wall of the CBD. MRI, magnetic resonance imaging; CBD, common bile duct.

**Figure 2:**
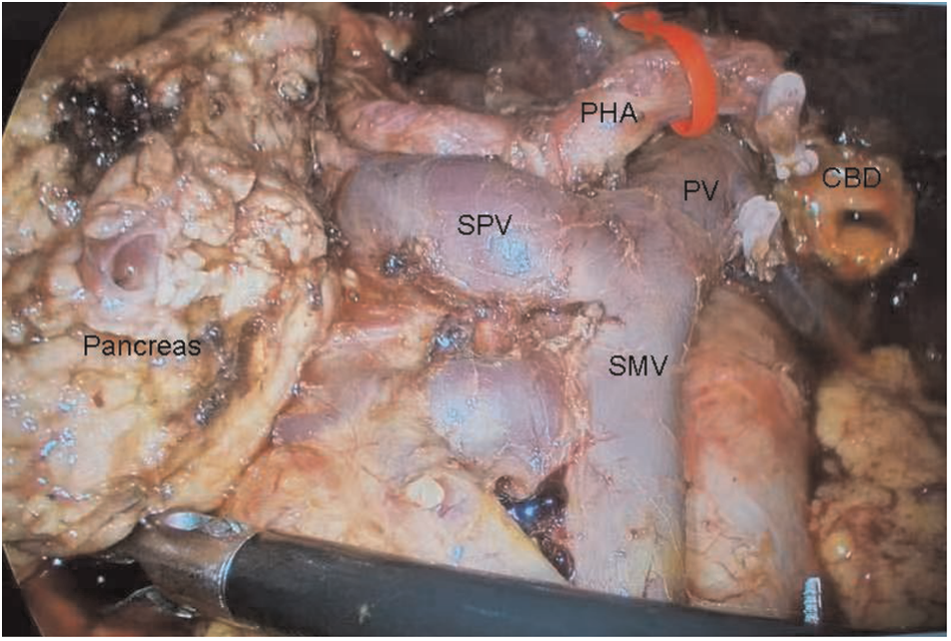
Regional anatomy in the laparoscopic pancreaticoduodenectomy. CBD, common bile duct; PHA, proper hepatic artery; PV, portal vein; SMV, superior mesenteric vein; SPV, splenic vein.

For the assistant examinations, the carbohydrate antigen 19-9 (CA 19-9) level was 126.4 units per milliliter (reference range, 0–27). Her blood total bilirubin was 230.1 μmol per liter (μmol/L) (reference range, 5.1–28.0). Magnetic resonance imaging (MRI) of the abdomen revealed the following: (1) SIT (Figures 1A,B); (2) distended gallbladder; (3) vine-tree-like dilatation of the intrahepatic and extrahepatic bile ducts (Figure 1C); (4) conspicuously cramped lower end of the common bile duct (CBD) and a thickened wall of the lower end of the CBD (Figure 1D); and (5) slight dilatation of the pancreatic duct. Therefore, cholangiocarcinoma was suspected in this simple SIT patient.

After admission to the hospital, the total bilirubin level was 376.5 μmol/L, and percutaneous transhepatic cholangial drainage (PTCD) was performed. The total bilirubin level decreased to 184.9 μmol/L. After the 20-day full preoperative preparation, the patient underwent LPD therapy (Figure 2).

The left-right transposition of viscera is greatly inconvenient for surgeons performing LPD, although the order of operative steps was similar. Therefore, in the first hour of the operation, the surgeon stood on the left side of the patient and spent substantial time observing the total left-right transposition of viscera, revising the surgical plan and practicing the possible operation of some surgical instruments. During LPD, the surgeon paid more attention to exploring the complex vascular variants one after another. The colon was located in front of the small bowel, and anastomosing the small intestine with the pancreas and bile duct were behind the colon. The cholangiojejunostomy and pancreaticojejunostomy were not performed until the surgeon had adapted to the patient's unique structure of left-right viscera transposition. The whole operation was successful and safe. The total bilirubin level was reduced to 98.1 μmol/L on the second day after the operation. The patient left the hospital and was lost to follow-up.

**Figure 3:**
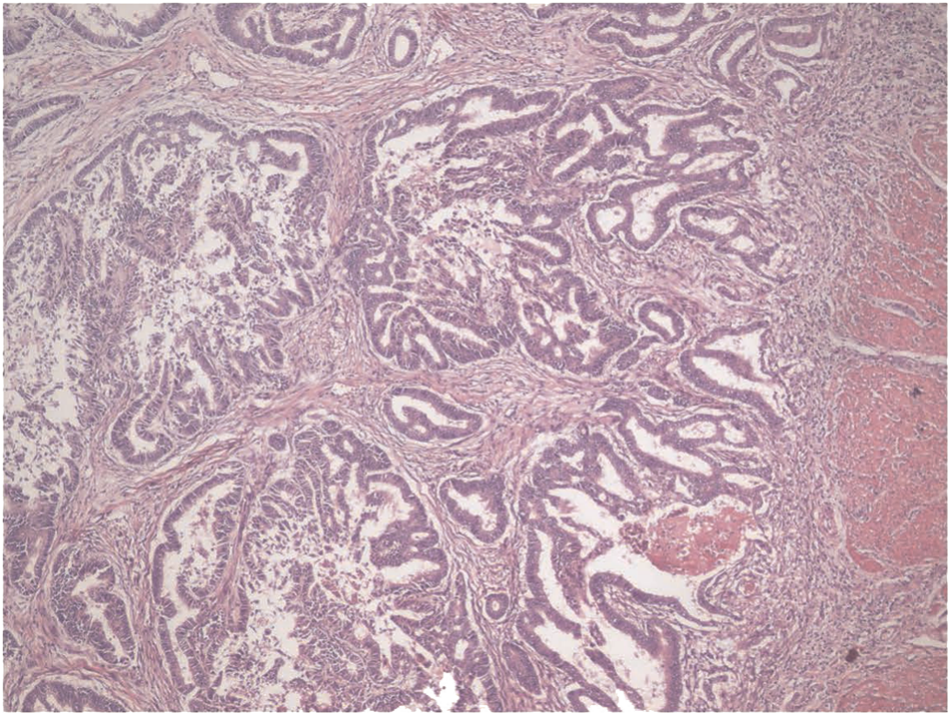
Histopathology specimens of adenocarcinoma of the duodenal papilla.

Finally, the results of histologic examination showed that the tumor was a moderately ulcerated and highly differentiated adenocarcinoma of the duodenal papilla (Figure 3), and tumor cells had invaded the CBD but failed to involve the pancreas tissues and lymph nodes.

## Discussion

A rare case was reported of a SIT patient with a suspected diagnosis of cholangiocarcinoma who was finally diagnosed with duodenal papillary adenocarcinoma after receiving successful LPD therapy.

The pathogenesis of SIT is unclear, and patients with SIT can be simple or complex. In this study, the patient presented with manifestations of chronic dacryocystitis and failed to be diagnosed with SIT at the first admission. This might be attributed to the fact that simple SIT does not directly have serious effects on people's health. It is noteworthy that SIT may be a part of multiple malformation syndromes, such as Kartagener's syndrome and Asplenia syndrome (5). These associated diagnoses in patients with SIT should not be ignored. Regarding the etiology of SIT, the improper development of embryonic tissues, especially the lateral plate mesoderm, may be the key cause (6). Family genetic predisposition might be irrelevant to SIT (4). In addition, the influence of gender on SIT was unclear. However, these results were concluded from case reports or studies with small samples. It will be very interesting to analyze the relationships between SIT, gender and family genetic predisposition in larger studies.

Recently, there were a few cases describing some SIT patients with cancer. In contrast to a previous case (7), the patient in this study showed elevated CA 19-9 levels and typical radiography features of cholangiocarcinoma. However, the histologic examination after LPD suggested adenocarcinoma of the duodenal papilla. The outcomes of different cancers might be completely different. The survival time of duodenal papilla adenocarcinoma patients might be longer than that of cholangiocarcinoma patients (8). In clinical practice, performing surgery to obtain histopathology specimens is the most effective and direct way to avoid misdiagnosis of cancer.

While the difficulty of performing LPD was substantial, SIT was not a contraindication. Full evaluation of regional anatomy and experienced surgeons standing in the appropriate positions during the operation were of great importance in reducing serious surgical complications and mortality (7). Unlike some patients with bowel malrotation (9), the patient in our study only presented a total left-right transposition of viscera, and the colon was still located in front of the small bowel. There was a surgical ergonomic change during the operation. For this patient with SIT, the surgeon stood on the left side of the patient to perform LPD. In the preoperative period, making a surgical plan based on imaging data was necessary, but revising surgical steps according to the regional anatomy during the operation was of vital importance for reducing surgical complications. It took a substantial amount of time to recognize the dangerous zone of dissection and perform the deceptively simple surgical steps in the context of this unique anatomical structure. With the help of artificial intelligence (AI), the increase in data from surgical videos of LPD could shorten the length of surgery and increase the safety of LPD. An intraoperative assistant tool should be developed in the future to provide real-time guidance to recognize the dangerous zone of dissection during LPD (10, 11).

Another key factor in successful LPD was careful management in the perioperative period. Improvement of patients’ status in the preoperative period is important, such as reducing the level of blood total bilirubin and increasing the albumin level. Intense postoperative monitoring should be performed, especially the features of cardiovascular function on electrocardiograms. In addition, to maintain the safety of patients, it is worth converting LPD into laparotomic operation when necessary. Transferring these patients to a large medical center for LPD would decrease the risk of complications after surgery and improve patient prognosis.

Increasing the discrimination power of imaging examination for different tumors is another method to avoid misdiagnosis of cancer. In this study, the results of radiography analysis were inaccurate, partly due to human error. Radiography data are objective, and the analysis of radiography data *via* AI may be a good way to narrow human error for avoiding misdiagnosis. A recent review reported the usage of AI to analyze surgical data, including intraoperative radiography data (11).

In conclusion, misdiagnosis of duodenal papilla adenocarcinoma was avoided in a patient with SIT *via* LPD. When cancer was suspected, it was possible to perform a safe LPD to obtain histopathology specimens, and it was a cost-effective method for avoiding misdiagnosis and directly implying the patient's prognosis.

## Data Availability

The raw data supporting the conclusions of this article will be made available by the authors, without undue reservation.
